# Data supporting possible implication of APOBEC2 in self-renewal functions of myogenic stem satellite cells: Toward understanding the negative regulation of myoblast differentiation

**DOI:** 10.1016/j.dib.2017.03.051

**Published:** 2017-04-08

**Authors:** Hideaki Ohtsubo, Yusuke Sato, Takahiro Suzuki, Wataru Mizunoya, Mako Nakamura, Ryuichi Tatsumi, Yoshihide Ikeuchi

**Affiliations:** a Department of Animal and Marine Bioresource Sciences, Graduate School of Agriculture, Kyushu University, Hakozaki, Fukuoka 812-8581, Japan; b Department of Bio-Productive Science, Utsunomiya University, Utsunomiya, Tochigi 321-8505, Japan; c Department of Molecular and Developmental Biology, Kawasaki Medical School, Kurashiki, Okayama 701-0192, Japan; d Graduate School of Agriculture, Kyushu University, Hakozaki, Fukuoka 812-8581, Japan

**Keywords:** APOBEC2, Myogenic stem satellite cells, Self-renewal, Muscle, Single-fiber culture, Differentiation

## Abstract

This paper provides *in vitro* phenotypical data to show that APOBEC2, a member of apoB mRNA editing enzyme, catalytic polypeptide-like family, may implicate in self-renewal functions of myogenic stem satellite cells, namely in the re-establishment of quiescent status after activation and proliferation of myoblasts in single-myofiber culture.

**Specifications Table**

**Table:** 

Subject area	*Biology*
More specific subject area	*Skeletal muscle biology, tissue-specific stem cell physiology*
Type of data	*Image (microscopy), graph*
How data was acquired	*Fluorescence Microscope (Leica DMI6000B fluorescence microscope equipped with a DFC365FX digital camera and LAS AF 3.1.0 software)*
Data format	*Raw (microscopy), analyzed (positive-cell counting)*
Experimental factors	*Single myofibers isolated from adult WT and APOBEC2-KO mice, cultured for 3 days in DMEM containing 10% normal horse serum and 0.5% chick embryo extract, and counted for Pax7/MyoD-positive cell % on fibers*
Experimental features	*Pax7/MyoD-immunofluorescence microscopy*
Data source location	*Fukuoka, Japan*
Data accessibility	*All relevant data are within the article*

**Value of the data**

• Resident myogenic stem satellite cell population observed here is a valuable target of research on postnatal muscle fiber growth, hyperplasia/hypertrophy, and regeneration after muscle injury.
• Molecular mechanism for myogenic cell fate determination, especially for “self-renewal” functions of satellite cells, is a big research subject and hence of value to the scientific community.
• APOBEC2 expression is predominant in skeletal and cardiac muscles and elevated exclusively at the early-differentiation phase of myoblasts in muscle regeneration; however the biological and physiological significance is still unknown.
• The particular idea of an essential role for APOBEC2 in the self-renewal functions may extend our understanding of the previous finding that APOBEC2 negatively drives regulation of myoblast differentiation and fusion (see Ref. [1]).

## 1 Data

**Fig. 1:**
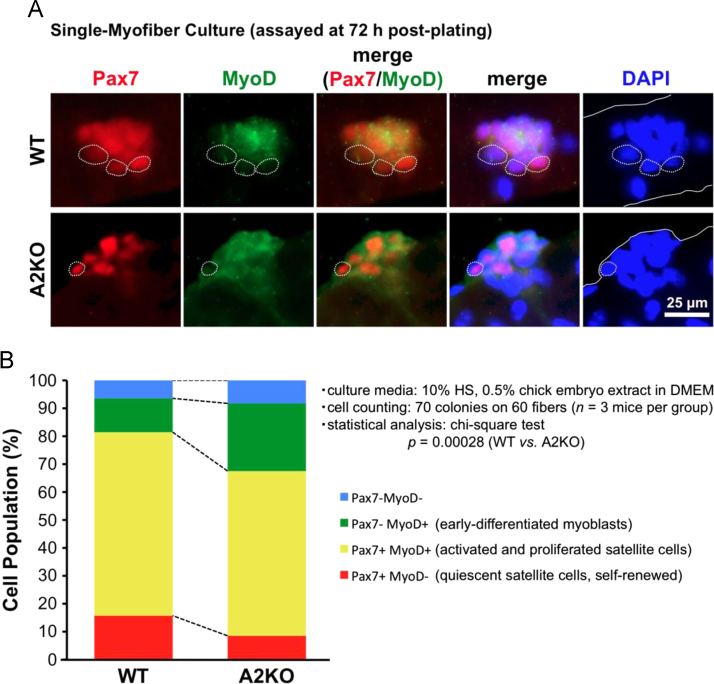
Effect of APOBEC2 deficiency on myogenic cell fate determination in single-myofiber culture. Single fibers were isolated from EDL muscle of adult male WT and A2KO mice and cultured for 72 h in DMEM containing 10% horse serum and 0.5% chick embryo extract followed by Pax7/MyoD-immunofluorescence microscopy (*n*=3 mice per group). (A) Representative views of satellite cell colonies on WT (upper row) and A2KO myofibers (lower row). Dotted-line circles, Pax7(+) MyoD(−) quiescent satellite cells concerned in this study; solid white-lines, outlines of myofiber. (B) Bars depict the mean and S.E. of each cell-population percentage. Note that Pax7(+) MyoD(−) cell percentage (*red bars*) significantly decreased along with a complementary increase in early-differentiated myoblast population (Pax7(−) MyoD(+) cells, *green bars*) on A2KO fibers (*p*<0.0005).

We tested a hypothesis that APOBEC2 may be an important mediator in the “self-renewal” functions of satellite cells, namely in the re-establishment of quiescent status after activation and proliferation. *in vitro* experiments in mouse single-myofiber cultures prepared from APOBEC2-KO (A2KO) mice demonstrated a significant decrease in the population of Pax7(+) MyoD(−) quiescent satellite cells along with a complementary increase in Pax7(−) MyoD(+) early-differentiated myoblasts concerned in Ref. [1] (*p*<0.0005) (Fig. 1), supporting a possible insight that APOBEC2 regulates a competitive balance between two trajectories of proliferated myoblasts during muscle regeneration: a return to cell quiescence which re-establishes the satellite cell pool and their differentiation and fusion which results in myotube formation.

## 2 Experimental design, materials and methods

### 2.1 Experimental design

To evaluate the above particular idea of a role for APOBEC2 in the self-renewal functions, single myofibers were isolated from wild-type (WT) and A2KO mice and assayed at 72 h post-plating for the population of Pax7(−) MyoD(−), Pax7(−) MyoD(+), Pax7(+) MyoD(+), and Pax7(+) MyoD(−) cells on fibers by immunofluorescence microscopy (see Fig. 1A).

### 2.2 Materials and methods

#### 2.2.1 Animal care and use

A2KO mice (C57BL/6 as the background strain) were generated by Dr. Neuberger (Medical Research Council Laboratory of Molecular Biology, United Kingdom) [2] and bred in our laboratory. Inbred C57BL/6 mice were used as WT controls. All animal experiments were conducted in strict accordance with the Guidelines for Proper Conduct of Animal Experiments published by the Science Council of Japan and ethics approvals from the Kyushu University Institutional Review Board (Approval nos. 20-12, 23-62, A22-218, A24-075, A26-078, and A28-090).

#### 2.2.2 Single-fiber isolation and culture

Single myofibers were isolated from extensor digitorum longus (EDL) muscle of 8-wk-old adult male WT and A2KO mice according to Anderson et al. [3] and Ravenscroft et al. [4] with some modifications [5], [6]. In brief, EDL muscle was dissected and digested with 0.2% (v/v) collagenase type 1 (CSL-1; Worthington Biochemical, Lakewood, NJ, USA) in high-glucose Dulbecco׳s modified Eagle׳s medium (DMEM) for 45 min at 37 °C. Muscles were triturated with a flame-polished Pasteur pipette to dissociate them into single muscle fibers, which were then maintained for 72 h in DMEM supplemented with 10% (v/v) normal horse serum (16050-122 from Invitrogen, Grand Island, NY, USA) and 0.5% (v/v) chick embryo extract in a humidified 5% CO_2_ atmosphere at 37 °C in an incubator followed by Pax7/MyoD-immunostaining.

#### 2.2.3 Double-immunofluorescence microscopy for satellite cell cluster analyses

Single myofibers were fixed at 72 h post-plating with 4% (v/v) paraformaldehyde (09154-85; Nacalai Tesque, Kyoto, Japan) in phosphate-buffered saline (PBS) for 5 min at room temperature. Fibers were quenched with 10 mM sodium citrate buffer (pH 6.8) and permeabilized with 0.5% (v/v) Triton X-100 in PBS followed by a blocking step with 20% normal goat serum (16210-064; Invitrogen) in PBS at room temperature for 1 h. Subsequently, fibers were incubated in a mixture of monoclonal anti-Pax7 (1:50 dilution in blocking solution; obtained from the Developmental Studies Hybridoma Bank, Iowa City, IA, USA) and polyclonal anti-MyoD antibodies (1:100 dilution; sc-760 from Santa Cruz Biotechnology, Santa Cruz, CA, USA) overnight at 4 °C. Alexa Fluor 594-labeled anti-mouse IgG (1:500 dilution in blocking solution; A21201, Invitrogen) and Alexa Fluor 488 anti-rabbit IgG (1:500 dilution; A-21441, Invitrogen) were used as secondary antibodies, respectively. Fibers were counter-stained with 4׳,6-diamidino-2-phenylindole (DAPI; 1:1000 dilution; D523 purchased from DOJINDO Laboratories, Kumamoto, Japan) and observed under a Leica DMI6000B fluorescence microscope equipped with a DFC365FX digital camera and LAS AF 3.1.0 software. Satellite cell clusters on fibers from WT and A2KO mice were analyzed for expression of Pax7 and MyoD (*n*=3 mice per group; 70 satellite cell colonies on 60 fibers, about 500 cells for each genotype); populations of Pax7(+) MyoD(−) and Pax7(−) MyoD(+) cells were assigned to quiescent satellite cells and early-differentiated myoblasts, respectively [7], [8].

#### 2.2.4 Statistical analysis

Chi-square tests were employed for statistical analysis of experimental results using Microsoft Excel X for Macintosh. Data are represented as mean±S.E. and the level of significance was set to *p*<0.05. Results are representative examples of at least three independent experiments.

## Funding sources

This work was funded by Grant-in-Aid for Scientific Research (B) 23380159 from the Japan Society for the Promotion of Science (JSPS) (to Y. Ikeuchi). Research was also supported, in part, by Grants-in-Aid for Scientific Research (A) 16H02585 and (B) 22380145 and 25292164, by the Invitation Fellowship Program for Research in Japan (JSPS), and by Grant funds from the Ito Foundation and Graduate School of Agriculture, Kyushu University (all to R. Tatsumi). H. Ohtsubo received a scholarship from Kyushu University during the course of this research.
